# Introducing a health information literacy competencies map: connecting the Association of American Medical Colleges Core Entrustable Professional Activities and Accreditation Council for Graduate Medical Education Common Program Requirements to the Association of College & Research Libraries Framework

**DOI:** 10.5195/jmla.2020.645

**Published:** 2020-07-01

**Authors:** Emily A. Brennan, Rikke Sarah Ogawa, Kelly Thormodson, Megan von Isenburg

**Affiliations:** 1 brennane@musc.edu, Research and Education Informationist, MUSC Libraries, and Associate Professor, Academic Affairs Faculty Department, MUSC Libraries, Medical University of South Carolina, Charleston, SC; 2 rikke.ogawa@library.ucla.edu, Director, Louise M. Darling Biomedical Library and Science and Engineering Library, University of California Los Angeles (UCLA), Los Angeles, CA; 3 kthormodson@pennstatehealth.psu.edu, Director, Harrell Health Sciences Library: Research and Learning Commons, Penn State University, Hershey, PA; 4 megan.vonisenburg@duke.edu, Associate Dean, Library Services & Archives, Duke University Medical Center, Durham, NC

## Abstract

**Background:**

Librarians teach evidence-based medicine (EBM) and information-seeking principles in undergraduate, graduate, and post-graduate medical education. These curricula are informed by medical education standards, medical education competencies, information literacy frameworks, and background literature on EBM and teaching. As this multidimensional body of knowledge evolves, librarians must adapt their teaching and involvement with medical education. Identifying explicit connections between the information literacy discipline and the field of medical education requires ongoing attention to multiple guideposts but offers the potential to leverage information literacy skills in the larger health sciences education sphere.

**Methods:**

A subgroup of the Association of Academic Health Sciences Libraries Competency-Based Medical Education Task Force cross-referenced medical education documents (Core Entrustable Professional Activities and 2017–2018 Liaison Committee on Medical Education Functions and Structures of a Medical School) with the Association of College & Research Libraries Framework for Information Literacy for Higher Education using nominal group technique.

**Results:**

In addition to serving as a vocabulary, the map can also be used to identify gaps between and opportunities for enhancing the scholarly expectations of undergraduate and graduate medical education standards and the building blocks of information literacy education.

## INTRODUCTION

Librarians teach evidence-based medicine (EBM) and information-seeking principles in undergraduate, graduate, and postgraduate medical education [1]. These curricula are informed by medical education standards [2], medical education competencies [3], information literacy frameworks [4], and background literature [5]. As this multidimensional body of knowledge evolves, librarians must adapt their teaching and involvement with medical education. Identifying explicit connections between the information literacy discipline and the medical education field requires ongoing attention to multiple guiding documents but offers the potential to leverage information literacy skills in the larger health sciences education sphere.

In 2015, at the recommendation of three academic medical education librarians from institutions in the Midwest and on the West Coast, the Association of Academic Health Sciences Libraries (AAHSL) formed a task force on librarians' roles and opportunities in competency-based medical education. Competency-based medical education is grounded in the idea that the time attached to learning (e.g., clerkship rotations) should not be the primary measure of educational achievement, but rather competence, as demonstrated through performance assessment of the trainee's competence in a specific area, should be [6]. The prior year, the Association of American Medical Colleges (AAMC) introduced the Core Entrustable Professional Activities (EPAs) and the associated Curriculum Developer's Guide [3]. EPAs are evolving frameworks of competency-based medical education that identify specific competencies that are expected of medical school graduates, regardless of future specialty. Their identification has prompted medical schools and librarians across the country to assess how their curricula prepare medical students to meet these expectations.

The task force was charged with two primary goals: to establish a baseline of library engagement in the Core EPAs and to create a cross-referencing map of existing EBM and information literacy documents [7, 8]. The cross-referencing map aligns the Association of College & Research Libraries (ACRL) Framework for Information Literacy for Higher Education, the Core EPAs, and the Accreditation Council for Graduate Medical Education (ACGME) Common Program Requirements. This paper explains the production of the cross-referencing map and its potential role as a tool to identify gaps and build vocabulary to advocate for increased librarian involvement in the continuum of medical education.

Furthermore, the task force's work built upon what was already known about librarian involvement in evidence-based components of undergraduate and graduate medical education curricula [1, 5] and provided an authoritative statement to present to organizations, such as AAMC and ACGME, to describe the contributions of health sciences librarians to evidence-based medical education and the new competencies.

### Association of College & Research Libraries (ACRL) Information Literacy Framework

In 2016, ACRL, a division of the American Library Association, developed a framework for information literacy for higher education. The ACRL Framework provides a structure and context through which to approach an information literacy curriculum. It is organized into six frames of interconnected core concepts of information literacy, rather than strict standards:

- “Authority Is Constructed and Contextual” (A): An author's expertise and credibility must be evaluated alongside the context in which the information was created and will be used.
- “Information Creation as a Process” (IC): Creation is an iterative process and results in a variety of information formats.
- “Information Has Value” (V): Information is a commodity with legal and social implications for its use.
- “Research as Inquiry” (RI): The research process is an iterative process of asking more complex questions.
- “Scholarship as Conversation” (S): Dialogue is necessary for the fullest interpretation of scholarship.
- “Searching as Strategic Exploration” (SC): Searching for information is a nonlinear process that requires mental flexibility and breadth of resources to understand concepts.

The frames contain specific knowledge practices that are intended to serve as “demonstrations of ways in which learners can increase their understanding of these information literacy concepts” [4]. The knowledge practices were selected by the task force to be the foundation of the map. Each frame has between six and eight associated knowledge practices, creating a total of forty-five elements to form the basis of the map. The knowledge practices were numbered according to their frames (e.g., SC-1 and RI-7).

### Core Entrustable Professional Activities (Core EPAs)

The Core EPAs are thirteen activities that all medical students should be able to perform upon entering residency, regardless of their future career specialties. EPAs are tasks or responsibilities that interns (first-year residents) perform unsupervised once they have attained sufficient and specific competence [3]. Each EPA has an associated set of critical functions that further break down the knowledge and skills needed to accomplish the activity.

The AASHL task force selected the three EPAs that were most relevant to information behaviors for mapping onto the ACRL Framework elements:

- EPA 7: “Form Clinical Questions and Retrieve Evidence to Advance Patient Care”
- EPA 9: “Collaborate as a Member of an Interprofessional Team”
- EPA 13: “Identify System Failures and Contribute to a Culture of Safety and Improvement”

The other ten EPAs were not considered for mapping because they focused on elements of clinical practice that had no direct relationship to information literacy instruction.

EPAs 7, 9, and 13 each have eight critical functions, which are critical activities to demonstrate competence, resulting in a set of twenty-four EPA elements to be mapped against the forty-five knowledge practice elements of the ACRL Framework. These functions were assigned numbers based on their positions in each EPA (e.g., EPA 7-2 and EPA 9-6) [3].

### Accreditation Council for Graduate Medical Education (ACGME) Common Program Requirements

The ACGME Common Program Requirements are the guiding standards used to evaluate programs, and train and prepare resident and fellow physicians in the graduate medical education continuum [9]. The document has six major areas of requirements that cover a program's institution, program personnel and resources, resident appointments, educational program, evaluation, and learning and work environment. The task force selected the specific standards mentioned in the ACGME Common Program Requirements for mapping to the ACRL Framework, based on their relevance to education and information behaviors and similarity to the selected Core EPAs:

- IV.A.5.c.: “Practice-based Learning and Improvement”
- IV.A.5.d.: “Interpersonal and Communication Skills”
- IV.A.5.e.: “Professionalism”
- IV.B.: “Residents' Scholarly Activities”
- VI.E.: “Clinical Responsibilities, Teamwork, and Transitions of Care”

Each of the ACGME Common Program Requirements listed above has three to eight narrower elements, resulting in a set of twenty-nine narrower elements that more discreetly define how programs should meet the standards. Because the ACGME Common Program Requirements vary in specificity within elements, the group mapped either the twenty-nine narrower elements or the five broader ACGME Common Program Requirements to map to the forty-five knowledge practices of the ACRL Framework, resulting in thirty-four potential components of ACGME Program Requirements.

With these documents and the task force charge in hand, the task force began mapping library professional standards for information literacy to newly emerging competencies from medical education to provide librarians with a lexicon for advocacy for librarians' role as educators and to translate information literacy frameworks into the competency-based medical standards that were common to medical educators. The cross-referencing map aligned the undergraduate and graduate medical education competencies and standards to the ACRL Information Literacy Framework, a gold standard document for many academic librarians. In addition to serving as a vocabulary, the map was designed to be used to identify gaps and opportunities for enhancing the scholarly expectations of undergraduate and graduate medical education standards and the building blocks of information literacy education.

## METHODS

The group originally planned to map the AAMC Core EPAs and the ACGME Common Program Requirements to the ACRL Framework in pairs, with two librarians working together to map each component of the ACRL Framework. However, agreement between mappers was very low in an initial test set of competencies, and it was clear that inter-rater reliability would not be achievable independently. To facilitate greater consensus, the method was changed to a methodology based on the Nominal Group Technique (NGT) [10]. NGT is a face-to-face consensus-building methodology involving 5 steps: (1) identification of the problem, (2) private generation of ideas, (3) recording of each group member's ideas, (4) facilitated group discussion of all ideas, and (5) voting and ranking. The mapping team consisted of four members dispersed across a large geographic distance. Therefore, the process was adapted to:

1. **Identification of the problem:** Selection and debate of relevant competency and standards included specific AAMC EPAs and ACGME Common Program Requirements that were relevant to information literacy education and practice.
2. **Private generation of ideas:** Independently and asynchronously, members mapped all selected EPAs and Common Program Requirements to the ACRL Framework on separate spreadsheets.
3. **Recording of each group member's ideas:** During regularly scheduled group meetings, group members added their mappings to one shared group map with columns for each group member, revealing their mappings to each other for the first time.
4. **Facilitated group discussion of all ideas:** The four members took turns moderating the discussion, which consisted of reading each element of the ACRL Framework and having each member of the group report which Core EPA or Common Program Requirement they mapped to it.
5. **Voting and Ranking:** In issues of complete agreement, the agreed upon EPA or Common Program Requirement and sub-item was added to the map. When there was any disagreement, the group discussed the potentially mapped items, using both the six broad ACRL frames and examples of practical applications of the knowledge practices in the health professions environment to determine appropriate mappings.

The group worked through all forty-five ACRL Framework knowledge practices until consensus was achieved for each.

After the AAMC Core EPAs were mapped to the ACRL Framework, the group repeated the process with the ACGME Common Program Requirements, mapping each of the thirty-four elements of the selected ACGME Common Program Requirements to the forty-five knowledge practices of the ACRL Framework as the group achieved consensus.

## RESULTS

### Core EPAs and the ACRL Framework: gaps and alignment

**Figure 1:**
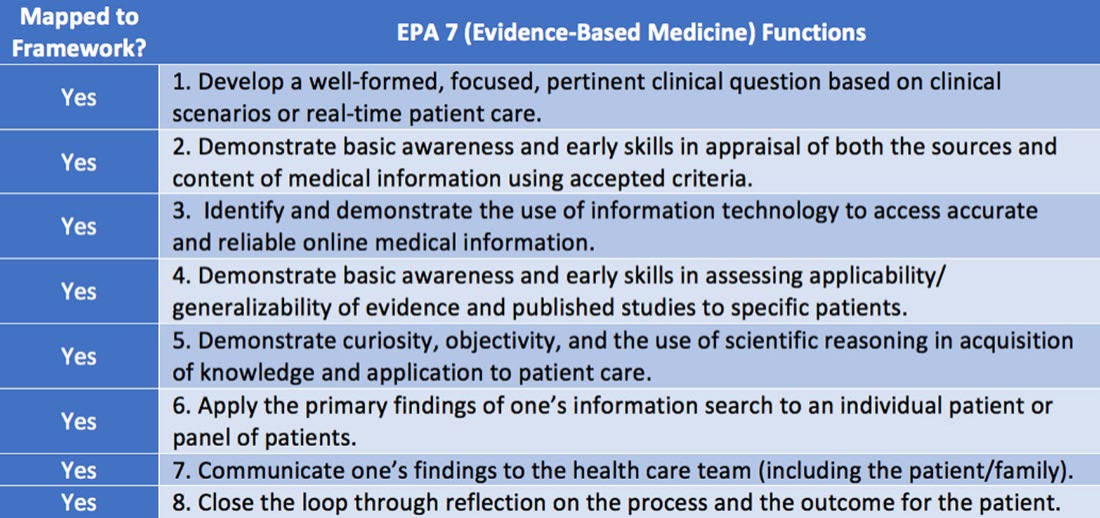
Core Entrustable Professional Activity (EPA) 7 mapped to the Association of College & Research Libraries (ACRL) Framework

Of the EPAs, only EPA 7 mapped to all six information literacy frames; EPAs 9 and 13 mapped to three of the six frames (Figure 1). Given the information literacy scope of the ACRL Framework, it was logical that more items from the EPA that were related to evidence-based practice were mapped relative to those items from the EPAs that were related to interprofessional teamwork, and safety and quality improvement.

The frames mapped as follows:

- “Authority Is Constructed and Contextual”: EPA 7, 9 and 13
- “Information Creation as a Process”: EPA 7
- “Information Has Value”: EPA 7, 9 and 13
- “Research as Inquiry”: EPA 7
- “Searching as Strategic Exploration”: EPA 7
- “Scholarship as Conversation”: EPA 7, 9 and 13

While all information literacy frames mapped to at least one EPA, some ACRL knowledge practices were not mapped to any Core EPAs (supplemental appendix). The most frequently mapped EPAs to the ACRL Framework included:

- EPA 7-2, “Demonstrate basic awareness and early skills in appraisal of both the sources and content of medical information using accepted criteria”
- EPA 7-3, “Identify and demonstrate the use of information technology to access accurate and reliable online medical information”

**Figure 2:**
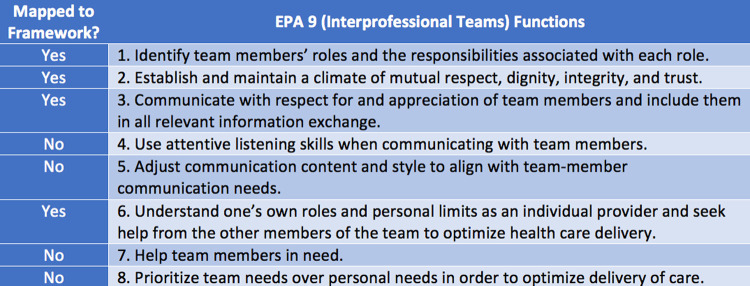
EPA 9 mapped to the ACRL Framework

**Figure 3:**
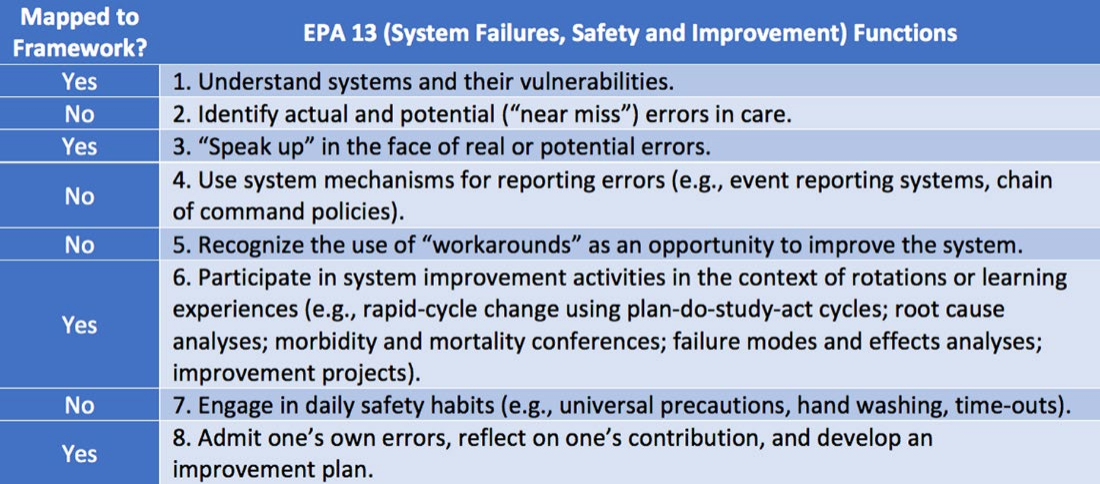
EPA 13 mapped to the ACRL Framework

**Figure 4:**
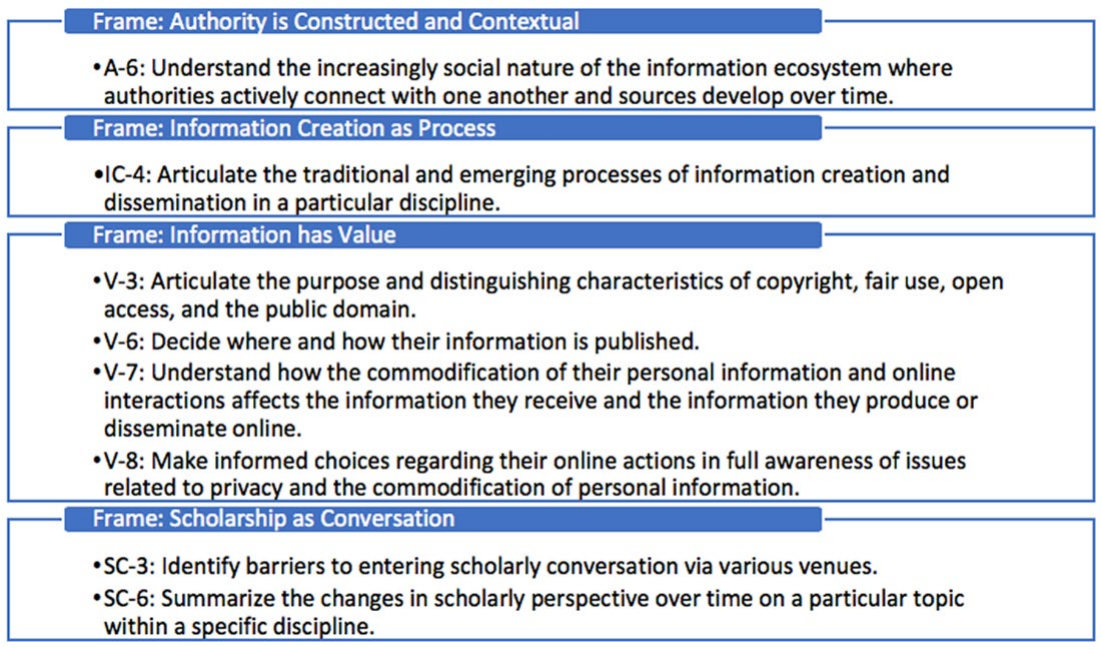
ACRL knowledge practices that did not map to the EPAs

All functions in EPA 7 (evidence-based medicine) mapped to the ACRL Framework, which was not the case for other EPAs. In both EPA 9 (interprofessional teams) (Figure 2) and EPA 13 (system failures, safety, and improvement) (Figure 3), four of eight critical functions mapped to the ACRL Framework. Critical functions that did not map related to principles of communication techniques and safety, rather than issues of information use. There were eight ACRL knowledge practices that did not map to the EPAs (Figure 4).

### ACGME Common Program Requirements and the ACRL Framework: gaps and alignment

The ACGME requirement that was most mapped to the ACRL Framework was IV.A.5.c, which refers to practice-based learning and improvement, specifically, “Residents must demonstrate the ability to investigate and evaluate their care of patients, to appraise and assimilate scientific evidence, and to continuously improve patient care based on constant self-evaluation and life-long learning.” The second most mapped requirement was IV.B., which is the broad category of “Residents' Scholarly Activities.”

In the ACGME requirements that were selected for mapping, the number of specific elements that mapped to specific knowledge practices varied greatly. While broad concepts of the ACGME Common Program Requirements implied competence in a frame in order to accomplish the element, the element itself might not have specific observable competencies that could be mapped. For example, requirement IV.A.5.d.: “Residents must demonstrate interpersonal and communication skills that result in the effective exchange of information” assumed the foundational ability to apply the frames of “Research as Inquiry,” “Authority Is Constructed and Contextual,” and “Scholarship as Conversation” in order to adequately apply the principles of EBM in dialogue with other professionals.

Five of six ACRL frames and their related knowledge practices were mapped from the selected ACGME requirements. “Information Creation as a Process” had no knowledge practices mapped from a Common Program Requirement.

## DISCUSSION

Mapping both the Core EPAs and the ACGME Common Program Requirements to the ACRL Framework presented numerous challenges. First among these were the differences in the purpose and structure of these three documents. The ACRL Framework was based on a group of interrelated core concepts, rather than on a rigid set of standards or skills. These conceptual understandings “organize many other concepts and ideas about information, research, and scholarship into a coherent whole” [4]. On the other hand, the AAMC Core EPAs and ACGME Common Program Requirements were prescriptive and defined discrete competencies that physicians should be able to perform and that medical education programs must teach.

While the mapping team chose to map at the most specific level possible using the ACRL Framework's knowledge practices, the AAMC EPAs' critical functions, and the narrowest elements of the ACGME Common Program Requirements, there were still challenges because of the varied scope and depth of the documents. The ACRL Framework, which focused on information literacy, went into greater detail than either the EPAs or the Common Program Requirements. It, therefore, became necessary to map some higher-level Common Program Requirements to the narrower knowledge practices, for example, ACGME IV.A.5.e, professionalism.

Finally, each member of the mapping team brought their own experiences and contexts to the process. All four members viewed these documents through the lenses of their institutions and their medical school curricula. Each member taught information literacy and EBM differently and at varying levels, based on their own institutional needs. That variety of experience made full agreement challenging, but also made the map more generalizable to other institutions.

The map reflects areas of high and low alignment between the AAMC EPAs and ACGME Common Program Requirements to the ACRL Framework. In general, the EPAs and Common Program Requirements do not contain the full breadth of information literacy practices reflected in the ACRL Framework. While this is logical given the unique purposes of all three documents, the authors propose that the gaps identified in the map can create opportunities for dialogue with health sciences librarians and medical educators.

Health sciences librarians and other medical education faculty should consider students' existing knowledge of information literacy practices. Many university librarians have implemented, or plan to implement, the ACRL Framework into their information literacy instruction [11, 12]. However, implementation of the ACRL Framework does not necessarily guarantee that students from adopting institutions will enter medical school with strong information literacy skills. Therefore, librarians and faculty should consider assessing students' baseline skills before launching an EBM curriculum. In addition, librarians should consider incorporating ACRL terminology from undergraduate information literacy instruction into curricular discussions with other faculty to draw explicit connections between information literacy and EBM, for example, stating that “Searching as Strategic Exploration is aligned with the *acquire* step of EBM.”

Nonlibrarian advocates for EBM in medical education should be encouraged to look to the ACRL Framework—not just the Core EPAs or Common Program Requirements—for a potentially broader set of related practices and knowledge that can enhance EBM curricula. Some knowledge practices that could augment the critical functions of EPA 7 include those related to the frames of “Information Has Value” and “Scholarship as a Conversation,” specifically issues of attribution, privacy, and barriers to joining the scholarly conversation. Many of these knowledge practices do not appear in the EPAs or Common Program Requirements and are, therefore, at risk of being left out of medical education. However, having an understanding of the ethical concerns of scholarship and the development of a physician's scholarly voice is implied in ACGME requirements that are related to residents' scholarly activities and could, therefore, be considered part of a continuum of information literacy education that is relevant to medical education.

The purpose of the map is to aid librarians in making connections between information literacy and medical education and to advocate for the inclusion of librarians across the continuum of medical education. In making these connections explicit between the ACRL Framework, the EPAs, and the ACGME requirements, librarians can use the map to clearly identify gaps in teaching and assessing information literacy at their own institutions. The information gained from local analyses can aid librarians' advocacy efforts with education leadership to ensure students are meeting these competencies. The authors encourage others to take up the challenge to map the educational competencies of additional disciplines in the health sciences to the ACRL Framework in order to allow broader advocacy efforts and to compare information literacy activities in other health-related disciplines.

## SUPPLEMENTAL FILE

**Appendix:** Health Information Literacy Competencies Map©
