# Supplementary material for: Introducing a health information literacy competencies map: connecting the Association of American Medical Colleges Core Entrustable Professional Activities and Accreditation Council for Graduate Medical Education Common Program Requirements to the Association of College & Research Libraries Framework
Source: J Med Libr Assoc. 2020 Jul 1;108(3):420–7. doi: 10.5195/jmla.2020.645 (PMC7441909; doi:10.5195/jmla.2020.645)
Supplement: Supplementary file 1 — Appendix: Health Information Literacy Competencies Map© [file jmla-108-3-420-s01.pdf]

JMLA Journal of the Medical Library Association

SC
Supplemental content to
J Med Libr Assoc. Jul;108(3):dx.doi.org/10.5195/jmla.2020.645
www.jmla.mlanet.org
© Brennan, Ogawa, Thormodson, von Isenburg 2020

Introducing a health information literacy competencies map: connecting the Association of American Medical Colleges Core Entrustable Professional Activities and Accreditation Council for Graduate Medical Education Common Program Requirements to the Association of College & Research Libraries Framework

Emily A. Brennan; Rikke Sarah Ogawa, AHIP; Kelly Thormodson; Megan von Isenburg, AHIP

APPENDIX

Health Information Literacy Competencies Map©

Association of College & Research Libraries (ACRL): Framework for Information Literacy for Higher Education [1]		Association of American Medical Colleges (AAMC): Core Entrustable Professional Activities (EPAs) for Entering Residency [2]		Accreditation Council for Graduate Medical Education (ACGME): Common Program Requirements [3]
**ACRL knowledge practice #**	**ACRL knowledge practice**	**EPA #**	**EPA functions**	**ACGME Core Competency and Common Program Requirement**
ACRL Frame: Authority Is Constructed and Contextual (A)				
A-1	Define different types of authority, such as subject expertise (e.g., scholarship), societal position (e.g., public office or title), or special experience (e.g., participating in a historic event).	7, 9	7-2: Appraise evidence (evidence-based practice [EBP])<br>9-1: Identify team members' roles (interprofessional teamwork [IP])	n/a
A-2	Use research tools and indicators of authority to determine the credibility of sources, understanding the elements that might temper this credibility.	7	7-2: Appraise evidence (EBP)	IV.A.5.c: Practice-based learning and improvement
A-3	Understand that many disciplines have acknowledged authorities in the sense of well-known scholars and publications that are widely considered "standard," and yet, even in those situations, some scholars would challenge the authority of those sources.	7	7-2: Appraise evidence (EBP)	IV.A.5.c: Practice-based learning and improvement

JMLA Journal of the Medical Library Association

Supplemental content to
J Med Libr Assoc. Jul;108(3):dx.doi.org/10.5195/jmla.2020.645
www.jmla.mlanet.org
© Brennan, Ogawa, Thormodson, von Isenburg 2020

Association of College & Research Libraries (ACRL): Framework for Information Literacy for Higher Education [1]		Association of American Medical Colleges (AAMC): Core Entrustable Professional Activities (EPAs) for Entering Residency [2]		Accreditation Council for Graduate Medical Education (ACGME): Common Program Requirements [3]
**ACRL knowledge practice #**	**ACRL knowledge practice**	**EPA #**	**EPA functions**	**ACGME Core Competency and Common Program Requirement**
A-4	Recognize that authoritative content may be packaged formally or informally and may include sources of all media types.	7	7-2: Appraise evidence (EBP)	n/a
A-5	Acknowledge they are developing their own authoritative voices in a particular area and recognize the responsibilities this entails, including seeking accuracy and reliability, respecting intellectual property, and participating in communities of practice.	9, 13	9-6: Understand limits and seek help (IP)<br>13-3: Communicate errors (safety)<br>13-6: Participate in system improvement activities (safety)<br>13-8: Reflect and improve (safety)	VI.A.1.b.3: Engagement in quality improvement activities<br>VI.E.2: Teamwork
A-6	Understand the increasingly social nature of the information ecosystem where authorities actively connect with one another and sources develop over time.	n/a	n/a	n/a
			This frame (A) mapped to 6 EPA functions.	*This frame (A) mapped to 3 requirements.*
ACRL Frame: Information Creation as Process (IC)				
IC-1	Articulate the capabilities and constraints of information developed through various creation processes.	7	7-2: Appraise evidence (EBP)	n/a
IC-2	Assess the fit between the information product's creation process and a particular information need.	7	7-2: Appraise evidence (EBP)	n/a

JMLA Journal of the Medical Library Association

Supplemental content to
J Med Libr Assoc. Jul;108(3):dx.doi.org/10.5195/jmla.2020.645
www.jmla.mlanet.org
© Brennan, Ogawa, Thormodson, von Isenburg 2020

**Association of College & Research Libraries (ACRL): Framework for Information Literacy for Higher Education [1]**		**Association of American Medical Colleges (AAMC): Core Entrustable Professional Activities (EPAs) for Entering Residency [2]**		**Accreditation Council for Graduate Medical Education (ACGME): Common Program Requirements [3]**
**ACRL knowledge practice #**	**ACRL knowledge practice**	**EPA #**	**EPA functions**	**ACGME Core Competency and Common Program Requirement**
IC-3	Articulate the traditional and emerging processes of information creation and dissemination in a particular discipline.	n/a	n/a	n/a
IC-4	Recognize that information may be perceived differently based on the format in which it is packaged.	7	7-2: Appraise evidence (EBP)	n/a
IC-5	Recognize the implications of information formats that contain static or dynamic information.	7	7-2: Appraise evidence (EBP)	n/a
IC-6	Monitor the value that is placed upon different types of information products in varying contexts.	7	7-3: Acquire information (EBP)<br>7-4: Assess generalizability of evidence (EBP)	n/a
IC-7	Transfer knowledge of capabilities and constraints to new types of information products.	7	7-3: Acquire information (EBP)	n/a
IC-8	Develop, in their own creation processes, an understanding that their choices impact the purposes for which the information product will be used and the message it conveys.	7	7-4: Assess generalizability of evidence (EBP)<br>7-5: Demonstrate scientific reasoning (EBP)<br>7-6: Apply evidence (EBP)<br>7-8: Reflect on process (EBP)	n/a
			This frame (IC) mapped to 6 EPA functions.	*This frame (IC) mapped to 0 requirements.*

JMLA Journal of the Medical Library Association

SC

Supplemental content to
J Med Libr Assoc. Jul;108(3):dx.doi.org/10.5195/jmla.2020.645
www.jmla.mlanet.org
© Brennan, Ogawa, Thormodson, von Isenburg 2020

**Association of College & Research Libraries (ACRL): Framework for Information Literacy for Higher Education [1]**		**Association of American Medical Colleges (AAMC): Core Entrustable Professional Activities (EPAs) for Entering Residency [2]**		**Accreditation Council for Graduate Medical Education (ACGME): Common Program Requirements [3]**
**ACRL knowledge practice #**	**ACRL knowledge practice**	**EPA #**	**EPA functions**	**ACGME Core Competency and Common Program Requirement**
		ACRL Frame: Information Has Value (V)		
V-1	Give credit to the original ideas of others through proper attributions and citation.	9	9-2: Establish respect and trust (IP)<br>9-3: Communicate with respect (IP)	IV.A.5.e: Professionalism
V-2	Understand that intellectual property is a legal and social construct that varies by culture.	9	9-2: Establish respect and trust (IP)<br>9-3: Communicate with respect (IP)	IV.A.5.e: Professionalism
V-3	Articulate the purpose and distinguishing characteristics of copyright, fair use, open access, and the public domain.	n/a	n/a	IV.A.5.e: Professionalism
V-4	Understand how and why some individuals or groups of individuals may be underrepresented or systematically marginalized by the systems that produce and disseminate information.	9, 13	9-3: Communicate with respect (IP)<br>13-1: Understand systems (safety)	IV.A.5.e.5: Sensitivity to diverse patient population<br>VI.A.1.b.1.a: Quality improvement education in health care disparities<br>VI.A.1.b.3.a.i: Quality improvement activities in health care disparities
V-5	Recognize issues of access or lack of access to information sources.	7, 13	7-3: Acquire information (EBP)<br>13-1: Understand systems (safety)	n/a
V-6	Decide where and how their information is published.	n/a	n/a	IV.B.2: Scholarly activity

JMLA Journal of the Medical Library Association

SC
Supplemental content to
J Med Libr Assoc. Jul;108(3):dx.doi.org/10.5195/jmla.2020.645
www.jmla.mlanet.org
© Brennan, Ogawa, Thormodson, von Isenburg 2020

**Association of College & Research Libraries (ACRL): Framework for Information Literacy for Higher Education [1]**		**Association of American Medical Colleges (AAMC): Core Entrustable Professional Activities (EPAs) for Entering Residency [2]**		**Accreditation Council for Graduate Medical Education (ACGME): Common Program Requirements [3]**
**ACRL knowledge practice #**	**ACRL knowledge practice**	**EPA #**	**EPA functions**	**ACGME Core Competency and Common Program Requirement**
V-7	Understand how the commodification of their personal information and online interactions affects the information they receive and the information they produce or disseminate online.	n/a	n/a	n/a
V-8	Make informed choices regarding their online actions in full awareness of issues related to privacy and the commodification of personal information.	n/a	n/a	IV.A.5.e.3: Respect for patient privacy
			This frame (V) mapped to 4 EPA functions.	*This frame (V) mapped to 6 requirements.*
ACRL Frame: Research as Inquiry (RI)				
RI-1	Formulate questions for research based on information gaps or on reexamination of existing, possibly conflicting, information.	7	7-1: Develop clinical question (EBP)	IV.A.5.c: Practice-based learning and improvement<br>IV.B: Scholarly activities
RI-2	Determine an appropriate scope of investigation.	7	7-1: Develop clinical question (EBP)	IV.A.5.c: Practice-based learning and improvement<br>IV.B: Scholarly activities
RI-3	Deal with complex research by breaking complex questions into simple ones, limiting the scope of investigations (e.g., problem, intervention, comparison, outcome [PICO]).	7	7-1: Develop clinical question (EBP)	IV.A.5.c: Practice-based learning and improvement<br>IV.B: Scholarly activities

JMLA Journal of the Medical Library Association

Supplemental content to
J Med Libr Assoc. Jul;108(3):dx.doi.org/10.5195/jmla.2020.645
www.jmla.mlanet.org
© Brennan, Ogawa, Thormodson, von Isenburg 2020

Association of College & Research Libraries (ACRL): Framework for Information Literacy for Higher Education [1]		Association of American Medical Colleges (AAMC): Core Entrustable Professional Activities (EPAs) for Entering Residency [2]		Accreditation Council for Graduate Medical Education (ACGME): Common Program Requirements [3]
**ACRL knowledge practice #**	**ACRL knowledge practice**	**EPA #**	**EPA functions**	**ACGME Core Competency and Common Program Requirement**
RI-5	Use various research methods, based on need, circumstance, and type of inquiry.	7	7-3: Acquire information (EBP)	IV.A.5.c: Practice-based learning and improvement<br>IV.B: Scholarly activities
	Monitor gathered information and assess for gaps or weaknesses.	7	7-2: Appraise evidence (EBP)<br>7-4: Assess generalizability of evidence (EBP)	IV.A.5.c: Practice-based learning and improvement<br>IV.B: Scholarly activities
RI-6	Organize information in meaningful ways.	7	7-7: Communicate findings (EBP)	IV.A.5.c: Practice-based learning and improvement<br>IV.B: Scholarly activities
RI-7	Synthesize ideas gathered from multiple sources and draw reasonable conclusions based on the analysis and interpretation of information.	7	7-4: Assess generalizability of evidence (EBP)	IV.A.5.c: Practice-based learning and improvement<br>IV.B: Scholarly activities
RI-8	Draw reasonable conclusions based on the analysis and interpretation of information.	7	7-5: Demonstrate scientific reasoning (EBP)<br>7-6: Apply evidence (EBP)	IV.A.5.c: Practice-based learning and improvement<br>IV.B: Scholarly activities
			This frame (RI) mapped to 7 EPA functions.	*This frame (RI) mapped to 2 requirements.*
ACRL Frame: Searching as Strategic Exploration (S)				
S-1	Determine the initial scope of the task required to meet their information needs.	7	7-1: Develop clinical question (EBP)	IV.A.5.c.3: Identify and perform appropriate learning activities

JMLA Journal of the Medical Library Association

Supplemental content to
J Med Libr Assoc. Jul;108(3):dx.doi.org/10.5195/jmla.2020.645
www.jmla.mlanet.org
© Brennan, Ogawa, Thormodson, von Isenburg 2020

Association of College & Research Libraries (ACRL): Framework for Information Literacy for Higher Education [1]		Association of American Medical Colleges (AAMC): Core Entrustable Professional Activities (EPAs) for Entering Residency [2]		Accreditation Council for Graduate Medical Education (ACGME): Common Program Requirements [3]
**ACRL knowledge practice #**	**ACRL knowledge practice**	**EPA #**	**EPA functions**	**ACGME Core Competency and Common Program Requirement**
S-2	Identify interested parties—such as scholars, organizations, governments, and industries—who might produce information about a topic and then determine how to access that information.	7	7-2: Appraise evidence (EBP)<br>7-3: Acquire information (EBP)	IV.A.5.c.6: Locate and appraise evidence<br>IV.A.5.c.7: Use information technology
S-3	Utilize divergent (e.g., brainstorming) and convergent (e.g., selecting the best source) thinking when searching.	7	7-2: Acquire information (EBP)	IV.A.5.c.6: Locate and appraise evidence
S-4	Match information needs and search strategies to appropriate search tools.	7	7-3: Acquire information (EBP)	IV.A.5.c.6: Locate and appraise evidence<br>IV.A.5.c.7: Use information technology
S-5	Design and refine needs and search strategies as necessary, based on search results.	7	7-3: Acquire information (EBP)	IV.A.5.c.6: Locate and appraise evidence<br>IV.A.5.c.7: Use information technology
S-6	Understand how information systems are organized to access relevant information.	7	7-3: Acquire information (EBP)	IV.A.5.c.7: Use information technology
S-7	Use different searching language types (e.g., Medical Subject Headings [MeSH], keywords, natural language).	7	7-3: Acquire information (EBP)	IV.A.5.c.6: Locate and appraise evidence<br>IV.A.5.c.7: Use information technology
S-8	Manage searching processes and results effectively.	7	7-3: Acquire information (EBP)	IV.A.5.c.7: Use information technology
			This frame (S) mapped to 3 EPA functions.	*This frame (S) mapped to 3 requirements.*

SC
Supplemental content to
J Med Libr Assoc. Jul;108(3):dx.doi.org/10.5195/jmla.2020.645
www.jmla.mlanet.org
© Brennan, Ogawa, Thormodson, von Isenburg 2020

Association of College & Research Libraries (ACRL): Framework for Information Literacy for Higher Education [1]		Association of American Medical Colleges (AAMC): Core Entrustable Professional Activities (EPAs) for Entering Residency [2]		Accreditation Council for Graduate Medical Education (ACGME): Common Program Requirements [3]
**ACRL knowledge practice #**	**ACRL knowledge practice**	**EPA #**	**EPA functions**	**ACGME Core Competency and Common Program Requirement**
		ACRL Frame: Scholarship as Conversation (SC)		
SC-1	Cite the contributing work of others in their own information production.	9	9-2: Establish respect and trust (IP)	IV.A.5.e: Professionalism
SC-2	Contribute to scholarly conversation at an appropriate level, such as local online community guided discussion, undergraduate research journal, conference presentation/poster session.	7, 13	7-7: Communicate findings (EBP)<br>13-6: Participate in system improvement activities (safety)	IV.B.2: Scholarly activity
SC-3	Identify barriers to entering scholarly conversation via various venues.	n/a	n/a	n/a
SC-4	Critically evaluate contributions made by others in participatory information environments.	7, 13	7-2: Appraise evidence (EBP)<br>13-3: Communicate errors (safety)	IV.A.5.d: Interpersonal and communication skills<br>IV.A.5.c.6: Locate and appraise evidence<br>VI.E.2: Teamwork
SC-5	Identify the contribution that particular articles, books, and other scholarly pieces make to disciplinary knowledge.	7, 9	7-4: Assess generalizability of evidence (EBP)<br>9-2: Establish respect and trust (IP)	n/a
SC-6	Summarize the changes in scholarly perspective over time on a particular topic in a specific discipline.	n/a	n/a	n/a

Journal of the Medical Library Association

Supplemental content to
J Med Libr Assoc. Jul;108(3):dx.doi.org/10.5195/jmla.2020.645
www.jmla.mlanet.org
© Brennan, Ogawa, Thormodson, von Isenburg 2020

Association of College & Research Libraries (ACRL): Framework for Information Literacy for Higher Education [1]		Association of American Medical Colleges (AAMC): Core Entrustable Professional Activities (EPAs) for Entering Residency [2]		Accreditation Council for Graduate Medical Education (ACGME): Common Program Requirements [3]
**ACRL knowledge practice #**	**ACRL knowledge practice**	**EPA #**	**EPA functions**	**ACGME Core Competency and Common Program Requirement**
SC-7	Recognize that a given scholarly work may not represent the only or even the majority perspective on the issue.	7	7-4: Assess generalizability of evidence (EBP)	IV.A.5.c: Practice-based learning and improvement
			This frame (SC) mapped to 6 EPA functions.	*This frame (SC) mapped to 6 requirements.*

Developed by the Association of Academic Health Sciences Libraries (AAHSL) Competency-Based Medical Education Task Force (2018).

The Association of Academic Health Sciences Libraries (AAHSL) Competency-Based Medical Education Task Force asserts the right to first publication of the Health Information Literacy Competencies Map.

1. Association of College & Research Libraries. Framework for information literacy for higher education [Internet]. American Library Association [cited 5 Jun 2018]. <http://www.ala.org/acrl/standards/ilframework>.
2. Association of American Medical Colleges. Core entrustable professional activities for entering residency: curriculum developers' guide [Internet]. The Association [cited 31 Jul 2018]. <https://store.aamc.org/downloadable/download/sample/sample_id/63>.
3. Accreditation Council for Graduate Medical Education. ACGME common program requirements [Internet]. The Council [cited 31 Jul 2018]. <http://www.acgme.org/Portals/0/PFAssets/ProgramRequirements/CPRs_2017-07-01.pdf>.
